# Progression of the canonical reference malaria parasite genome from 2002–2019

**DOI:** 10.12688/wellcomeopenres.15194.2

**Published:** 2019-05-28

**Authors:** Ulrike Böhme, Thomas D. Otto, Mandy Sanders, Chris I. Newbold, Matthew Berriman

**Affiliations:** 1 Parasite Genomics, Wellcome Sanger Institute, Hinxton, Cambridge, CB10 1SA, UK; 2 Institute of Infection, Immunity and Inflammation, MVLS, University of Glasgow, Glasgow, G12 8QQ, UK; 3 Weatherall Institute of Molecular Medicine, University of Oxford, John Radcliffe Hospital, Oxford, OX3 9DU, UK

**Keywords:** Plasmodium, falciparum, genome, reference, annotation, curation

## Abstract

Here we describe the ways in which the sequence and annotation of the *Plasmodium falciparum* reference genome has changed since its publication in 2002. As the malaria species responsible for the most deaths worldwide, the richness of annotation and accuracy of the sequence are important resources for the *P. falciparum *research community as well as the basis for interpreting the genomes of subsequently sequenced species. At the time of publication in 2002 over 60% of predicted genes had unknown functions. As of March 2019, this number has been significantly decreased to 33%. The reduction is due to the inclusion of genes that were subsequently characterised experimentally and genes with significant similarity to others with known functions. In addition, the structural annotation of genes has been significantly refined; 27% of gene structures have been changed since 2002, comprising changes in exon-intron boundaries, addition or deletion of exons and the addition or deletion of genes. The sequence has also undergone significant improvements. In addition to the correction of a large number of single-base and insertion or deletion errors, a major miss-assembly between the subtelomeres of chromosome 7 and 8 has been corrected. As the number of sequenced isolates continues to grow rapidly, a single reference genome will not be an adequate basis for interpreting intra-species sequence diversity. We therefore describe in this publication a population reference genome of *P. falciparum*, called Pfref1. This reference will enable the community to map to regions that are not present in the current assembly. *P. falciparum *3D7 will continue to be maintained, with ongoing curation ensuring continual improvements in annotation quality.

## Introduction

The genome of *Plasmodium falciparum* 3D7 (a clone from the NF54 ( Walliker *et al*., 1987) isolate), the species responsible for the most severe form of malaria, was the first reference genome published to support *Plasmodium* research. Its publication more than almost two decades ago ( Gardner *et al*., 2002) was a milestone, the impact of which is reflected in several thousand citations that mention the *P. falciparum* 3D7 genome. The sequencing of *P. falciparum* was initially accompanied by the draft genome of a rodent malaria species, *P. yoelii* ( Carlton *et al.*, 2002). These genomes were followed by those of several other *Plasmodium* spp, sequenced using Sanger sequencing technology, including human-infective species *P. vivax* ( Carlton *et al*., 2008), the monkey and human malaria parasite *P. knowlesi* ( Pain *et al*., 2008) and further rodent *Plasmodium* spp ( Hall *et al*., 2005). With the advent of much cheaper short-read technology, many more genomes have been sequenced, including the chimpanzee parasite *P. reichenowi* ( Otto *et al*., 2014), the monkey malaria parasites *P. cynomologi* ( Tachibana *et al*., 2012), *P. coatneyi* ( Chien *et al*., 2016), *P. inui* and *P. fragile*, the murine parasite *P. vinkei*, the human parasites *P. malariae*, *P. ovale* ( Rutledge *et al*., 2017) and the avian malaria parasites *P. gallinaceum* and *P. relictum* ( Böhme *et al*., 2018). Although, many of these genomes are highly fragmented draft assemblies, algorithms that use high coverage of aligned short reads have enabled a variety of cost-effective genome-assembly improvements for several species ( Swain *et al*., 2012).

*P. falciparum* 3D7 is a major focus of malaria research and the accuracy of its reference genome and annotation are vital for accelerating hypothesis-driven research. Moreover, the availability of a reference genome has additional importance: it underpins genome comparisons, across the suite of genome sequences that are now available for multiple *Plasmodium* species, and the global efforts to analyse genome variation amongst thousands of clinical and lab isolates. The need for a commitment to maintain and improve this genome has long been recognized by the Wellcome Sanger Institute. Through careful manual curation, highly accurate predictions of coding and non-coding genes have been added. Functional descriptions of genes have also been kept up to date, to reflect the growing volume of *P. falciparum* related scientific literature. In many ways the depth of annotation is similar to that more commonly associated with model organisms. For instance, Gene Ontology terms have been manually selected that capture from the scientific literature the richness of gene roles in a format that can be easily queried or used for inference in genome-wide analyses. Recent examples include the genome-wide analysis of transcriptional dynamics ( Painter *et al*., 2018) and the uncovering of common functions in essential genes ( Zhang *et al*., 2018).

Genome improvement and curation has resulted in thousands of individual changes over more than 15 years. In particular, the resolution of subtelomeric regions has been transformed along with the ability to annotate important multigene families that are often found in those regions.

This is the first paper to describe the changes since the *P. falciparum* 3D7 genome was first published. Originally 5,268 protein-coding genes were annotated and of those, over 60% (3,465 genes) of predicted genes had unknown functions ( Table 1). Despite the fact that there still seems to be a common perception that over 50% of genes remain functionally unannotated ( Briquet *et al*., 2018; Tang *et al*., 2019) the number of predicted genes has risen to 5,438 and the proportion without ascribed functions has now shrunk to 33% (1,776 genes) ( Table 1). Since 2002, 27% of genes have undergone structural changes or have been added based on RNAseq data and other data from publications. New ncRNAs have also been added and complete apicoplast and mitochondrial genomes have been assembled. One of the many purposes of a reference genome is to interpret natural variation data. In the latest version, we have therefore included alternative contigs representing major haplotypic differences. This reference dataset has been named Pfref1 to reflect the fact that it does not simply comprise *P. falciparum* 3D7 data but has been supplemented with other reference data to better represent the pan-genome for this species. The aim of Pfref1 is to enable robust mapping to analyse genome variation in regions of *Plasmodium* genomes where the current Pf3D7 genome (v3.2) is an unsuitable reference.

**Table 1.** Table showing the main differences between *P. falciparum* 3D7 version 1 (2002), version 2 (2005), version 2.1.4 (2007) and version 3.2 (March 2019). *Numbers includes partial genes, pseudogenes are not included. ** Nucleotide ambiguities present in genome v1 and v2 have been changed to gaps. ***Pseudogenes defined here are based on gene predictions that contain at least one frame shift or premature stop codon.

	2002 (version 1)	2005 (version 2)	2007 (version 2.1.4)	2019 (version 3.2)
**Nuclear genome**				
Genome size (bp)	22,853,764	23,289,065	23,264,337	23,292,622
Gaps	93	10	160 **	0
Genes *	5268	5414	5387	5280
Pseudogenes ***	NA	70	73	158
ncRNAs	0	0	606	103
Hypothetical proteins	3465	3545	3099	1776
tRNAs	43	27	44	45
**Apicoplast genome**				
Assembly size (kb)	Absent	Absent	Absent	34250
genes	Absent	Absent	Absent	30
**Mitochondrial genome**				
Assembly size	Absent	Absent	Absent	5967
genes	Absent	Absent	Absent	3

## Methods

### Curation and annotation

Changes to the genome annotation reflect ongoing work at the Wellcome Sanger Institute. The software Artemis (version 10 to version 18) was adapted to use a CHADO database schema ( Carver *et al*., 2008) and has been used continuously for manual curation and annotation. This database system is directly connected to GeneDB. Every 4 to 6 months data is transferred to PlasmoDB. To update functional annotation, Pubmed was searched (search terms *Plasmodium* and apicomplexa) on a regular basis for publications related to *Plasmodium*. Relevant information, i.e. gene product descriptions, EC numbers, gene names and functional descriptions to be captured by Gene Ontology terms, was extracted and changes manually added in Artemis. RNA-Seq data and TBLASTX comparisons were the primary supporting evidence for manual improvements to gene models. Information from user comments that were submitted to gene record pages in PlasmoDB were assessed and where relevant used to update annotation. Evidence codes that support product descriptions are available as GFF format genome annotation files from the following FTP site: ftp://ftp.sanger.ac.uk/pub/genedb/releases/latest/Pfalciparum/. To find annotation differences between genome versions we’ve performed pair-wise TBLASTX comparisons using the Artemis Comparison Tool (ACT) (version 13) ( Carver *et al*., 2005).

### DNA sequencing and correction of the *P. falciparum* 3D7 genome

After publication of the genome in 2002, manual finishing continued. Sequence changes and gap closure between genome versions 1 and 2 have been described ( Berry *et al*., 2004). Using a combination of capillary sequencing, Illumina sequencing, Pacific Biosciences sequencing and automated sequence correction, we have continued to improve the reference genome. Sequence errors on all chromosomes were corrected using the iCORN algorithm (version 1) ( Otto *et al*., 2010a). Two ambiguous regions towards the telomeres of chromosomes 7 and chromosome 8 were resolved using a PCR tiling path. The tiling path spanned the region between genes PF3D7_0805400 (MAL8P1.200) to PF3D7_0831200 (MAL8P1.204) on chromosome 8 and from PF3D7_0833500 (MAL7P1.212) to PF3D7_0701900 (PF07_0004) on chromosome 7. From the results of these the assembly of the left hand side of chromosome 8 was confirmed. There was a gap in chromosome 7 over a highly repetitive region of ^~^20kb. Read pair information from a 3kb insert Illumina library was used to identify unassembled contigs that could close the gap and PCR was used to confirm the assembly. Unassembled contigs were also searched for any unique coding sequence. One contig was found and this was linked to a region on the right hand side of chromosome 8 by Illumina read pairs. This was also confirmed by PCR. For genome version 3.1, the complete apicoplast was included. Resolving this sequence has been described ( Hunt *et al*., 2015). For genome version 3.2, the *P. falciparum* 3D7 mitochondrion was also included. This mitochondrion was part of a recently described PacBio assembly of *P. falciparum* 3D7 ( Otto *et al*., 2018). The mitochondrion was corrected using the iCORN algorithm ( Otto *et al*., 2010a) followed by circlator ( Hunt *et al*., 2015).

### PfRef1 reference genome

The *P. falciparum* 3D7 version 3.2 assembly was compared with PacBio assemblies that we have recently described for several other isolates ( Otto *et al*., 2018) to create a population reference that we have termed PfRef1. BLASTN comparisons of 3 lab isolates ( *P. falciparum* IT, *P. falciparum* DD2, *P. falciparum* HB3) were inspected together using ACT software ( Carver *et al*., 2005). Excluding single nucleotide polymorphisms and small insertions and deletions, major sequence differences were identified, manually extracted and are provided as small EMBL files. Each file contains a sequence variant with a 500 bp anchor sequence on either side to enable unambiguous placement along the 3D7 reference. The files are available on the following FTP site: ftp://ftp.sanger.ac.uk/pub/project/pathogens/Plasmodium/falciparum/PfRef1

## Results

### Improvement of the *P. falciparum* 3D7 assembly

An update to the published version of the *P. falciparum* 3D7 genome was released in 2005 (version 2), this included gap closure and completion of many chromosomes from telomere to telomere ( Table 1) ( Berry *et al*., 2004). In September 2011, version 3 of the genome was released that included the correction of two major miss-assemblies on chromosome 7 and 8, the replacement of all “N”s in the genome with corrected sequence and the correction of hundreds of sequencing errors. These improvements were brought about largely due to the availability of Illumina technology and the development of a genome correction algorithm ( Otto *et al*., 2010a). In addition, gene locus identifiers were changed to bring them into line with guidelines published by the European Nucleotide Archive (ENA): systematic identifiers were changed to start with the prefix PF3D7 followed by an underscore and the chromosome number. All previous identifiers were retained as searchable synonyms.

Version 3.1 included a complete apicoplast genome with a length of 34 kb. In the original genome project that was published in 2002 the *P. falciparum* 3D7 apicoplast was not sequenced. For the re-annotation process the apicoplast from *P. falciparum* isolate C10 was temporarily used (Genbank X95275.2, X95276.2) ( Wilson *et al*., 1996) and included in genome version 2.1.4. The published apicoplast from isolate C10 consisted of two large contigs that could not be assembled due to a large deletion caused by an almost identical inverted repeat. As previously reported ( Hunt *et al*., 2015), version 3.1 of the genome includes a complete apicoplast genome that includes the resolved 5kb repeat.

For the reannotation process the mitochondrial genome with Genbank ID M76611 was included. This Genbank entry only reports the source as *P. falciparum*. Our recently described *P. falciparum* 3D7 PacBio assembly ( Otto *et al*., 2018) contained a mitochondrial genome. Comparing this PacBio genome with M76611 revealed a difference of two bases: a SNP at base 772 (T to C) and at base 4952 (C to T). The SNP at base 772 is non-synonymous and results in an isoleucine to valine substitution in COX3 (PF3D7_MIT01400) at amino acid 250. The genome version with the correct mitochondrial genome has been named version 3.2.

### Manual annotation of *P. falciparum* 3D7

**Figure 1.**
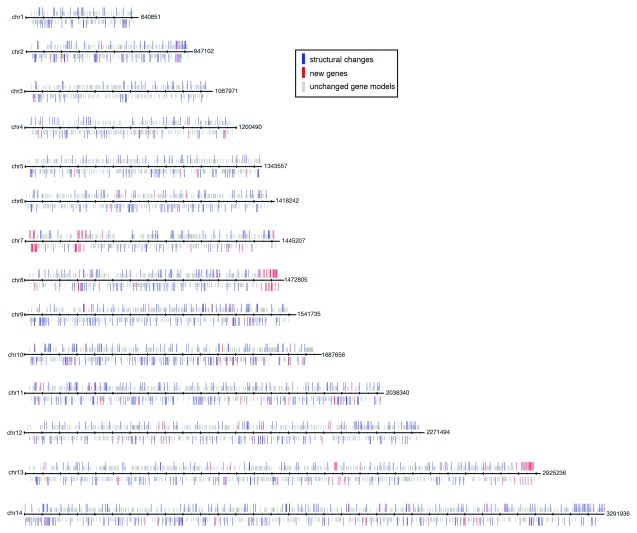
Distribution of genes with structural changes and new genes on chromosomes 1 to 14 of *P. falciparum* 3D7. The positions of new genes (shown in red), genes that have undergone structural changes (shown in blue) and genes that stayed the same since 2002 (shown in grey) are shown on the 14 chromosomes. The values along the right of each chromosome indicate the total sequence length in base pairs. Genes above the chromosome lines are located on the forward strand, genes below the chromosome lines are on the reverse strand.

**Figure 2.**
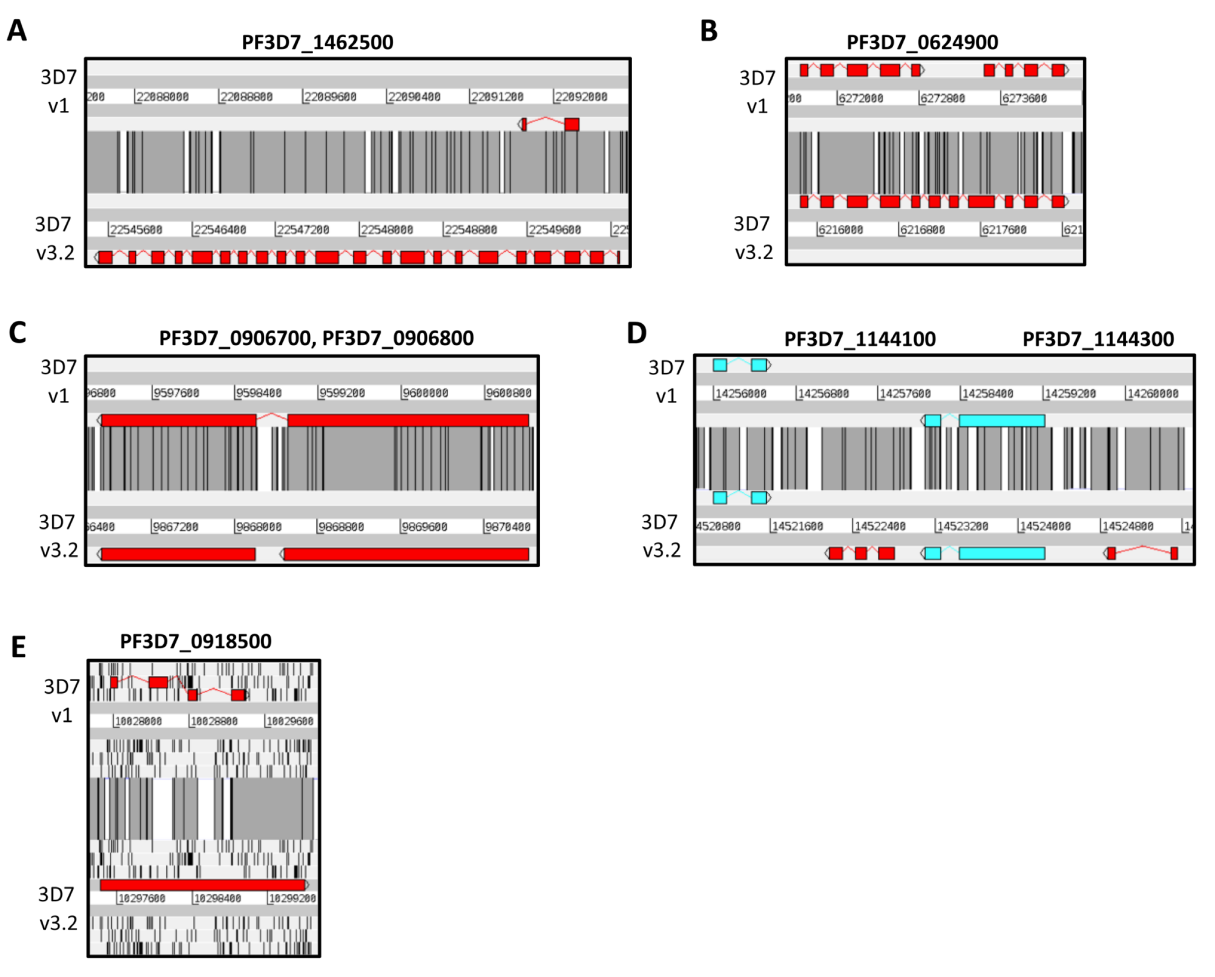
Gene structure changes. Artemis Comparison Tool (ACT) screenshot showing a comparison between 2002 and 2019. Coloured boxes represent genes. The grey blocks between sequences represent sequence similarity (TBLASTX). ( **A**) A 2-exon gene has been changed into a 22-exon gene (PF3D7_1462500) ( **B**) Two genes that have been merged (PF3D7_0624900) ( **C**) A gene that has been split into two genes (PF3D7_0906800, PF3D7_0906700) ( **D**) Two genes shown in red have been added (PF3D7_1144100, mitochondrial large subunit ribosomal protein; PF3D7_1144300, 60S ribosomal protein L41) ( **E**) A hypothetical gene (PFI0905w) has been deleted and a ncRNA (PF3D7_0918500, telomerase RNA) has been added. In (E), the six reading frames are shown with tick marks indicating stop codons.

**Figure 3.**
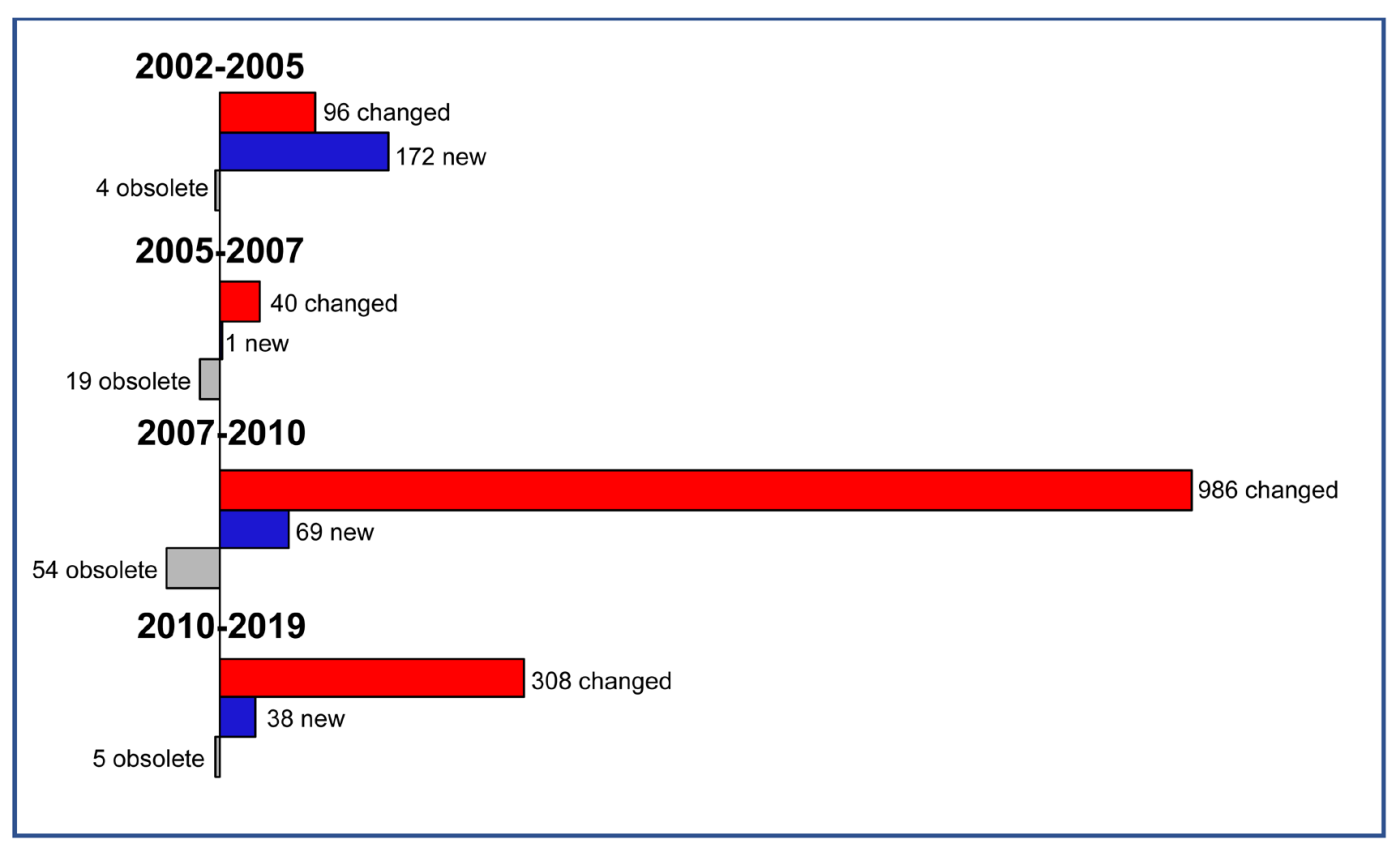
Diagram showing gene structure changes. Number of genes that have been added, deleted or changed are shown over four different time frames: October 2002 (genome version 1) and 2005 (genome version 2), between 2005 (version 2) and September 2007 (version 2.1.4), between September 2007 (genome version 2.1.4) and February 2010 (version 2.1.4) and between February 2010 and March 2019 (version 3.2). The number of changed genes includes gene models that have been merged, split or had a deletion/addition of exons or change of exon boundaries.

At the start of the *P. falciparum* 3D7 genome project manual gene finding was heavily reliant on sequence composition, especially GC content differences, due to an absence of alignable evidence. Over time additional *Plasmodium* genomes became available, in 2005 the rodent malaria genomes ( Hall *et al*., 2005) and in 2008 *P. knowlesi* ( Pain *et al*., 2008) and *P. vivax* ( Carlton *et al*., 2008). Having these additional *Plasmodium* genomes enabled gene structures to be revisited. A comprehensive reannotation process began with a workshop in 2007, involving approximately 40 members of the *Plasmodium* research community (see Box 1). One of the goals of the workshop was to ascribe updated functions to predicted proteins, check gene structures and systematically revisit the nomenclature for large gene families. A major new addition to the evidence was genome wide TBLASTX comparisons between species that were used to highlight conserved regions at the protein level and therefore identify positionally conserved orthologues and refine their exon-intron boundaries. In 2010, we published the first RNA-Seq data for this species ( Otto *et al*., 2010b). These data were used to further evaluate gene models and improve the accuracy of gene structures. As a result, 27% of genes have been added or had their structure changed since 2002 ( Figure 1). 1255 genes had changes to exon-intron boundaries or exons added or removed ( Figure 2A); this number include genes that were merged ( Figure 2B) or split ( Figure 2C). Since 2002, 244 genes have been added ( Figure 2D, Extended data: Table 1 ( Böhme, 2019)) and 36 predicted genes have been deleted due to a lack of evidence supporting their earlier prediction in regions of repetitive or unusual sequence, or because later RNAseq evidence (including strand-specific information) suggested that they were ncRNAs rather than protein-coding ( Figure 2E, Extended data: Table 2 ( Böhme, 2019)). In addition, a number of genes were created after 2002 based on algorithmic predictions but subsequently deleted due to a lack of supporting evidence (Extended data: Table 3 ( Böhme, 2019)). Figure 3 shows the number of changes to predicted gene structures, as well as the addition and deletion of new genes at four different time points.

**Table 2.** Gene Ontology (GO) annotation of *P. falciparum* 3D7 (version 3.1, 14.02.2019). The number of manually curated experimentally verified GO terms, manually curated terms that are based on sequence similarity and GO terms based on automated searches are listed ( Ashburner *et al.*, 2000; The Gene Ontology Consortium, 2017). IDA, inferred from direct assay; IPI, inferred from physical interaction; IMP, inferred from mutant phenotype; IGI, inferred from genetic interaction; HAD, inferred from high throughput direct assay; ISS, inferred from sequence or structural similarity; ISO, inferred from sequence orthology; ISM, inferred from sequence model; RCA, Reviewed computation analysis; IEA, inferred from electronic annotation. The IEA evidence code is either based on InterPro ( Jones *et al.*, 2014) or based on Chitale *et al.*, 2009 ( Chitale *et al.*, 2009). The GO annotation for 2002 was taken from ( Gardner *et al.*, 2002).

Gene Ontology	Number of GO annotations (2019)	Number of GO annotations (2002)
**Cellular Component**	6437	2,412
Experimental evidence code: IDA, IPI, IMP, IGI	1,867	
Evidence code based on sequence similarity: ISS, ISO, ISM	1,187	
Evidence code: RCA	175	
Evidence code: HDA	962	
Evidence code: IEA (InterPro)	719	
Evidence code: IEA (PMID:19435743)	1527	
**Molecular Function**	4,921	1,244
Experimental evidence code: IDA, IPI, IMP, IGI	979	
Evidence code based on sequence similarity: ISS, ISO, ISM	1,111	
Evidence code: IEA (InterPro)	1,732	
Evidence code: IEA (PMID:19435743)	1,099	
**Biological Process**	3,803	1,301
Experimental evidence code: IDA, IMP, IGI	857	
Evidence code based on sequence similarity: ISS, ISO, ISM	1,270	
Evidence code: IEA (InterPro)	1,117	
Evidence code: IEA (PMID:19435743)	559

Box 1. ***Plasmodium falciparum* community reannotation workshop**

A *Plasmodium falciparum* community reannotation workshop, co-organised by EuPathDB (David Roos) and GeneDB (Matthew Berriman) took place at the Wellcome Genome Campus Conference Center in Hinxton in October 2007. During this week-long workshop several hundred gene products were changed, including the annotation of ApiAP2 proteins.

Workshop participants:

Oliver Billker, Serge Bonnefoy, Pete Bull, Jane Carlton, Brendan Crabb, Hernando Del Portillo, Christian Doerig, Malcolm Gardner, Hagai Ginsburg, George Githinji, Aravind Iyer, Taco Kooij, Dominic Kwiatkowski, Sue Kyes, Thomas Lavstsen, Manuel Llinás, Eric Marechal, Dan Milner, Fingani Mphande, Dan Neafsey, Stuart Ralph, Gowthaman Ramasamy, Dhanasekaran Shanmugam, Robert Sinden, Worachart Sirawaraporn, Dominique Soldati, Tim Stedman, Xin-zhuan Su, Tom Templeton, Akhil Vaidya, Scott Westenberger and Jennifer Wortman.

Facilitators during the workshop included:

Andrew Berry, Céline Carret, Al Ivens, Arnab Pain, Adrian Tivey, Brian Brunk, Zhongqiang Chen, Mark Heiges and Lucia Peixoto.

Maintenance and setup of the Artemis Chado instance:

Tim Carver, Adrian Tivey, Chinmay Patel and Robin Houston.

New non-coding RNAs have been annotated based on transcriptome data, examples published in the scientific literature ( Chakrabarti *et al*., 2007; Guizetti *et al*., 2016; Raabe *et al*., 2010) and new predictive models in Rfam ( Kalvari *et al*., 2018). In 2007, 603 automatically predicted ncRNAs were included in the annotation ( Table 1) ( Mourier *et al*., 2008). Since 2002 the amount of aligned functional genomics data has increased enormously for this species providing deeper evidence support for most genuine transcripts. However, due to a lack of supporting evidence, the majority of previously predicted ncRNAs were subsequently removed from the reference annotation. Currently there are 103 ncRNAs annotated (Extended data: Table 2 ( Böhme, 2019)), including a recently described spliced antisense ncRNA that acts as a negative regulator (PF3D7_0935390), ( Filarsky *et al*., 2018).

**Figure 4.**
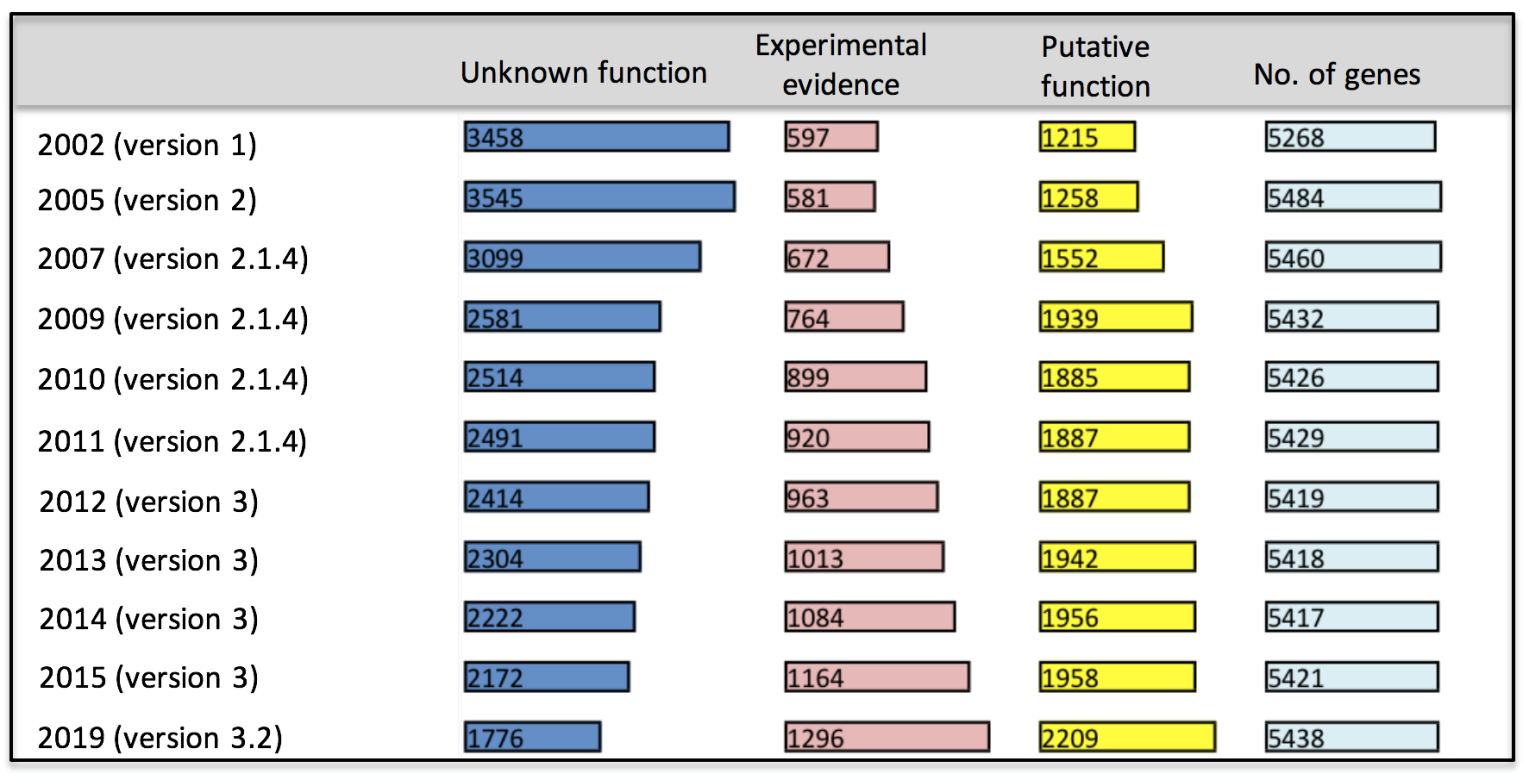
*P. falciparum* 3D7 annotation changes between October 2002 and March 2019. The number of genes between October 2002 and March 2019 are compared. The total number of genes includes pseudogenes. The number of genes with unknown function is shown (blue), genes with experimental evidence (red), genes with putative function (yellow) and the complete number of genes (light blue). Genes with unknown function have the following product description: conserved Plasmodium protein, unknown function; conserved protein, unknown function; conserved Plasmodium membrane protein, unknown function; Plasmodium exported protein, unknown function; probable protein, unknown function; hypothetical protein.

Using a combination of manual and automated methods, functional annotation has been radically improved since the initial publication in 2002. Revised annotation is based on a combination of literature searching, comments received from the research community, InterPro ( Jones *et al*., 2014) and sequence-similarity searches. Using these approaches the number of proteins with unknown function has almost halved, from 60% in 2002 to 33% in March 2019 ( Figure 4). The number of experimentally verified genes has changed from 597 genes in 2002 to 1296 in 2019 and the number of genes with putative functions has risen from 1215 to 2206. The richness of the annotation is reflected in the number of Gene Ontology (GO) terms ( Ashburner *et al*., 2000; The Gene Ontology Consortium, 2017) that have been manually assigned to genes based on published experiments, reflected in the following evidence codes: Inferred from direct assay (IDA), physical interaction (IPI), mutant phenotype (IMP) and genetic interaction (IGI). Altogether there are 1302 genes annotated using GO and supported by experimental evidence: 1095 genes captured by the “component” aspect of GO; 609 captured by “molecular function” and 369 genes captured by “biological process”. Because individual genes have been annotated with multiple terms, the number of individually curated and experimentally verified GO terms is much higher. There are 1867 GO components annotated, 979 GO functions and 857 GO processes. The manual GO annotation also includes 342 protein binding interactions ( Table 2). Annotation is updated continuously as new literature is published.

Throughout the annotation improvement process, engagement of the malaria research community has played an important role. The process started with the workshop in 2007 ( Box 1) but has continued through the activities of a dedicated full time curator aided by direct feedback and through comments that can be added by the community to gene record pages at PlasmoDB. These comments are constantly being evaluated and incorporated where relevant. The ongoing annotation is physically housed at the Sanger Institute, with updates regularly passed on to PlasmoDB (every 4 to 6 months).

### Population reference genome Pfref1

**Figure 5.**
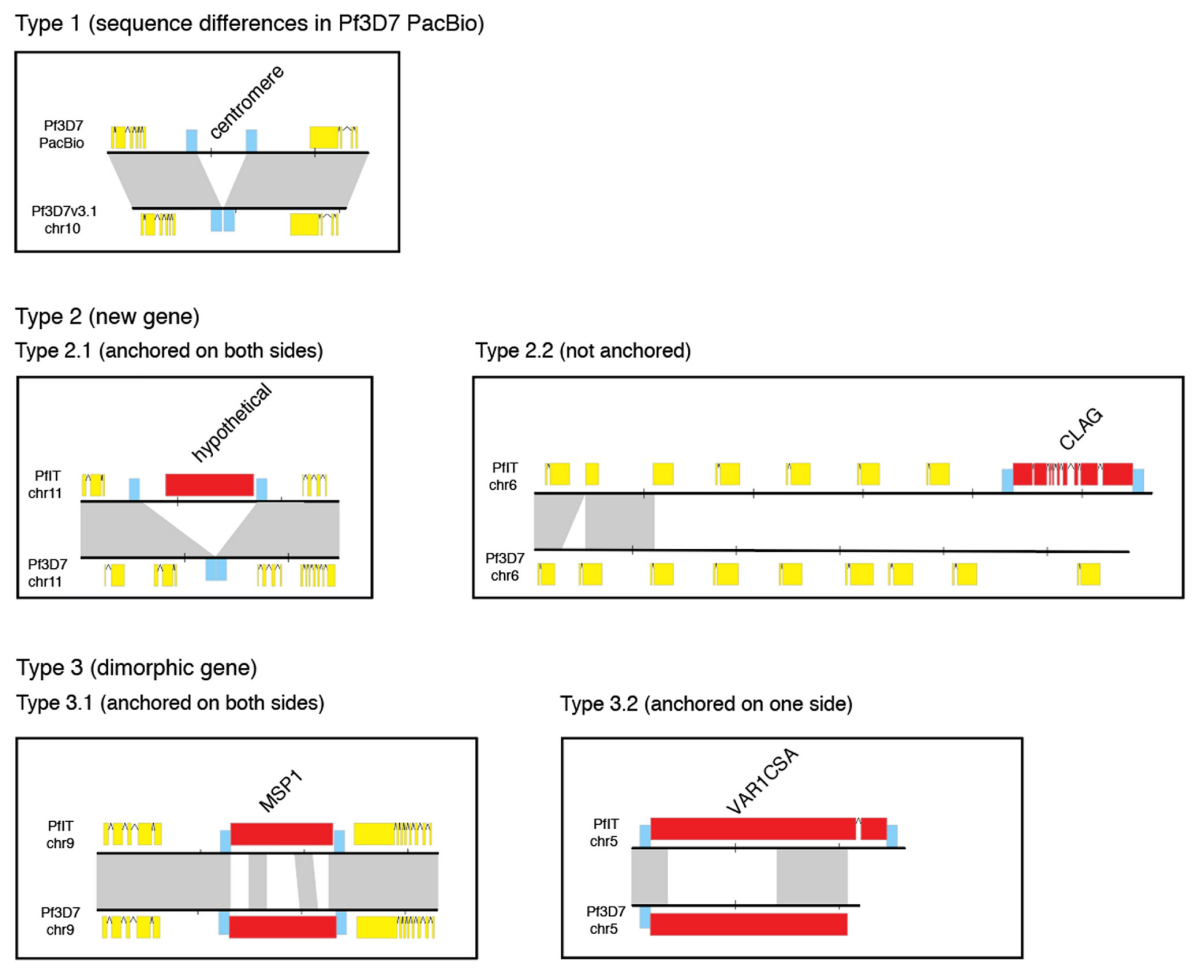
Diagram showing different types of patches created for the *P. falciparum* 3D7 population reference (PfRef1). Type-1 are sequence differences between the current *P. falciparum* 3D7 assembly version 3.2 and a new Pf3D7 PacBio assembly. 500 bp are provided on each side as anchor (shown in blue). Type-2 are new genes, that are either anchored on both sides (type 2.1), or not anchored (type 2.2). Type-3 are dimorphic genes that are either anchored on both sides (type 3.1) or anchored on one side (type 3.2).

**Figure 6.**
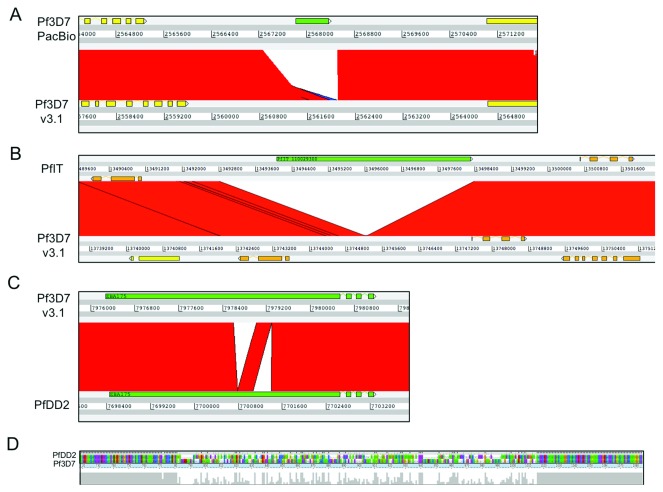
Differences between *P. falciparum* 3D7 genome version 3.2, a PacBio assembly of *P. falciparum* 3D7 and two lab strains *P. falciparum* IT and DD2. ACT comparisons between regions of the above genomes. Coloured boxes represent genes. The red blocks between sequences represent sequence similarity (BLASTn). ( **A**) In the current *P. falciparum* 3D7 genome assembly v3.2, a hypothetical protein on chromosome 13 is missing. This gene is present in a Pf3D7 PacBio assembly (shown in green). ( **B**) Comparison between *P. falciparum* IT chromosome 11 and *P. falciparum* v3.2 chromosome 11. *P. falciparum* 3D7 is missing a hypothetical gene on chr11. This gene is present in *P. falciparum* IT (PfIT_110029300) (shown in green). ( **C**) Comparison between Pf3D7 v3.2 chromosome 7 and *P. falciparum* DD2 chromosome 7. The comparison shows the dimorphic gene EBA175 erythrocyte binding antigen-175 (PF3D7_0731500). ( **D**) Clustalx alignment of EBA175 from PfDD2 and Pf3D7. The area shown is the dimorphic part of the two genes.

One of the many purposes of a reference is to interpret natural variation data, the aim being to enable robust mapping of re-sequencing reads from subsequent isolates. In the latest version we have incorporated sequence differences derived from 3 lab isolates assembled *de novo* as part of a collection of 15 PacBio reference genomes ( Otto *et al*., 2018). The differences have been incorporated into the reference as three classes ( Figure 5). The first (type-1) are “patches” to correct errors in Pf3D7 (version 3.2), for example a missing centromere on chromosome 10 and a missing gene on chromosome 13 ( Figure 5, Figure 6A), the second (type-2) are core genes that are present in other sequenced isolates, i.e. *P. falciparum* IT or *P. falciparum* DD2 but are missing in Pf3D7 ( Figure 5, Figure 6B) and the third (type-3) are dimorphic genes where alternative alleles cannot be mapped to the one currently present in Pf3D7 ( Figure 5, Figure 6C, Figure 6D). In total, there are now 17 type-1, four type-2 and 17 type-3 patches. The type-2 patches include genes encoding gamete associated protein (GAP), CLAG and hypothetical proteins. Type-3 include dimorphic genes encoding DBL-containing protein (PF3D7_0113800), Surfin 1.2, Surfin 8.3, Surfin 13.1, Surfin 14.1, MSP1, MSP2, MSP3, MSP6, S-antigen, EBA175, CLAG3.1, CLAG3.2, DBLMSP, DBLMSP2, CSP and VAR1CSA. For the new population reference, EMBL files containing the different types of patches including a 500 bp alignment on each side of the patch are provided. To reflect the fact that changes to 3D7 as well as data from other isolates have been included, we have termed the new reference sequence Pfref1.

## Discussion

In this paper we have provided an overview of the sequence and annotation changes the *P. falciparum* 3D7 genome has undergone since the initial publication in 2002. The inclusion of long-read sequencing has been critically important for spanning gaps that persisted for years in the reference assembly due to their extreme AT-richness and length. A previous attempt to produce an improved *P. falciparum* 3D7 reference assembly used Pacific Biosciences sequencing data assembled *de novo* ( Vembar *et al*., 2016). Although the assembly contiguity metrics were impressive, the authors did not attempt error-correction. As a consequence, a high proportion of gene sequences contained frameshifts and there were many unresolved repetitive sequences. In the present study, we have used automated error-correction assisted by a high coverage of aligned short reads, plus extensive manual review of individual read alignments. This has enabled us to drive the accuracy of underlying reference sequence, bringing a range of benefits. First, end users interested in individual genes have access to the most up to date information. Second, users interested in high throughput functional genomics or genome variation need the most up to date and complete sequence for mapping purposes to be available and used by all labs. Third, detailed curation in *P. falciparum* has a knock on effect across other important species of the genus because functional insights from one species can be projected to others based on homology. It is inevitable that there is a law of diminishing returns. However, with 33% of genes still of unknown function it is essential that ongoing maintenance, annotation and curation are continued. In particular, our future plans include the annotation of untranslated regions (UTRs) and the annotation of additional common alternative splice-forms for genes. We also plan to provide better visibility for evidence codes that support protein descriptions but are currently only available as GFF format genome annotation files. New possibilities are now being explored for the community to get involved with annotation. GeneDB will soon provide an opportunity for the community to contribute directly to structural annotation. Equivalent to that of an annotator’s view, the user will be able to view the curated *Plasmodium* genomes in Apollo ( Lee *et al*., 2013), a collaborative genomic annotation editor which allows multiple users to access the data.

The genetic structure of *P. falciparum* populations is currently studied by aligning whole genome sequencing reads to the Pf3D7 reference ( Manske *et al*., 2012; Neafsey *et al*., 2012). However, the approach has limitations: genomic regions that are absent or extremely diverged from 3D7 can not be analysed by alignment. This applies to genes that are completely missing as well as alleles of genes that are extremely different (either due to high sequence diversity or dimorphism). By supplementing *P. falciparum* 3D7 v3.2 with alternative loci we have created a combined new reference called Pfref1. Utilising such alternative loci, rather than simply excluding them from population analyses, remains a challenge and will require the further development of variant-calling methods. However, as a first step, the popular short-read alignment tool BWA-MEM ( Li, 2013), used in the GATK variant call pipeline, has been able to perform alignments in an alternative-aware mode for several years.

## Data availability

### Underlying data

*P. falciparum* 3D7 is maintained in GeneDB ( http://www.genedb.org) and updates are passed on at regular intervals to PlasmoDB ( http://www.plasmodb.org).

The annotation (GFF) files are being extracted from the annotation database on a monthly basis. They are available on the following FTP site:

ftp://ftp.sanger.ac.uk/pub/genedb/releases/latest/Pfalciparum/

The latest version of the database at time of publication is available here: 

ftp://ftp.sanger.ac.uk/pub/genedb/releases/2019-03/Pfalciparum/

The patches for the population reference are on the following FTP site.

ftp://ftp.sanger.ac.uk/pub/project/pathogens/Plasmodium/falciparum/PfRef1

This page also includes an explanation of the naming of the short EMBL files and instructions on how to map them to the reference genome.

### Extended data

Open Science Framework: Progression of the canonical reference malaria parasite genome from 2002–2019, https://doi.org/10.17605/OSF.IO/5K9VJ ( Böhme, 2019)

This project contains the following extended data:

- Table_1.xlsx ( *P. falciparum* 3D7 genes present in 2019 that were missing in 2002)
- Table_2.xlsx (List of ncRNAs present in *P. falciparum* 3D7 version 3.2)
- Table_3.xlsx ( *P. falciparum* 3D7 genes that were deleted anytime between 2002 and 2019)

Data are available under the terms of the Creative Commons Zero "No rights reserved" data waiver (CC0 1.0 Public domain dedication).

## Software availability

The Artemis software used for annotation is available on GitHub: http://sanger-pathogens.github.io/Artemis/Artemis/

## References

[ref-1] Ashburner M Ball CA Blake JA: Gene ontology: tool for the unification of biology. The Gene Ontology Consortium. *Nat Genet.* 2000;25(1):25–29. 10.1038/75556 10802651 PMC3037419

[ref-2] Berry AE Gardner MJ Caspers GJ: Curation of the *Plasmodium falciparum* genome. *Trends Parasitol.* 2004;20(12):548–552. 10.1016/j.pt.2004.09.003 15522662

[ref-3] Böhme U: Progression of the canonical reference malaria parasite genome from 2002–2019.2019 10.17605/OSF.IO/5K9VJ PMC6484455 31080894

[ref-4] Böhme U Otto TD Cotton JA: Complete avian malaria parasite genomes reveal features associated with lineage-specific evolution in birds and mammals. *Genome Res.* 2018;28(4):547–560. 10.1101/gr.218123.116 29500236 PMC5880244

[ref-5] Briquet S Ourimi A Pionneau C: Identification of *Plasmodium falciparum* nuclear proteins by mass spectrometry and proposed protein annotation. *PLoS One.* 2018;13(10):e0205596. 10.1371/journal.pone.0205596 30379851 PMC6209197

[ref-6] Carlton JM Adams JH Silva JC: Comparative genomics of the neglected human malaria parasite *Plasmodium vivax*. *Nature.* 2008;455(7214):757–763. 10.1038/nature07327 18843361 PMC2651158

[ref-39] Carlton JM Angiuoli SV Suh BB: Genome sequence and comparative analysis of the model rodent malaria parasite *Plasmodium yoelii yoelii*. *Nature.* 2002;419(6906):512–9. 10.1038/nature01099 12368865

[ref-7] Carver T Berriman M Tivey A: Artemis and ACT: viewing, annotating and comparing sequences stored in a relational database. *Bioinformatics.* 2008;24(23):2672–2676. 10.1093/bioinformatics/btn529 18845581 PMC2606163

[ref-8] Carver TJ Rutherford KM Berriman M: ACT: the Artemis Comparison Tool. *Bioinformatics.* 2005;21(16):3422–3423. 10.1093/bioinformatics/bti553 15976072

[ref-9] Chakrabarti K Pearson M Grate L: Structural RNAs of known and unknown function identified in malaria parasites by comparative genomics and RNA analysis. *RNA.* 2007;13(11):1923–1939. 10.1261/rna.751807 17901154 PMC2040097

[ref-10] Chien JT Pakala SB Geraldo JA: High-Quality Genome Assembly and Annotation for *Plasmodium coatneyi*, Generated Using Single-Molecule Real-Time PacBio Technology. *Genome Announc.* 2016;4(5): pii: e00883-16. 10.1128/genomeA.00883-16 27587810 PMC5009967

[ref-11] Chitale M Hawkins T Park C: ESG: extended similarity group method for automated protein function prediction. *Bioinformatics.* 2009;25(14):1739–1745. 10.1093/bioinformatics/btp309 19435743 PMC2705228

[ref-12] Filarsky M Fraschka SA Niederwieser I: GDV1 induces sexual commitment of malaria parasites by antagonizing HP1-dependent gene silencing. *Science.* 2018;359(6381):1259–1263. 10.1126/science.aan6042 29590075 PMC6219702

[ref-13] Gardner MJ Hall N Fung E: Genome sequence of the human malaria parasite *Plasmodium falciparum*. *Nature.* 2002;419(6906):498–511. 10.1038/nature01097 12368864 PMC3836256

[ref-14] Guizetti J Barcons-Simon A Scherf A: Trans-acting GC-rich non-coding RNA at *var* expression site modulates gene counting in malaria parasite. *Nucleic Acids Res.* 2016;44(20):9710–9718. 10.1093/nar/gkw664 27466391 PMC5175341

[ref-15] Hall N Karras M Raine JD: A comprehensive survey of the *Plasmodium* life cycle by genomic, transcriptomic, and proteomic analyses. *Science.* 2005;307(5706):82–86. 10.1126/science.1103717 15637271

[ref-16] Hunt M Silva ND Otto TD: Circlator: automated circularization of genome assemblies using long sequencing reads. *Genome Biol.* 2015;16:294. 10.1186/s13059-015-0849-0 26714481 PMC4699355

[ref-17] Jones P Binns D Chang HY: InterProScan 5: genome-scale protein function classification. *Bioinformatics.* 2014;30(9):1236–1240. 10.1093/bioinformatics/btu031 24451626 PMC3998142

[ref-18] Kalvari I Nawrocki EP Argasinska J: Non-Coding RNA Analysis Using the Rfam Database. *Curr Protoc Bioinformatics.* 2018;62(1):e51. 10.1002/cpbi.51 29927072 PMC6754622

[ref-19] Lee E Helt GA Reese JT: Web Apollo: a web-based genomic annotation editing platform. *Genome Biol.* 2013;14(8):R93. 10.1186/gb-2013-14-8-r93 24000942 PMC4053811

[ref-20] Li H: Aligning sequence reads, clone sequences and assembly contigs with BWA-MEM. *arXiv:13033997v2.* 2013 Reference Source

[ref-21] Manske M Miotto O Campino S: Analysis of *Plasmodium falciparum* diversity in natural infections by deep sequencing. *Nature.* 2012;487(7407):375–379. 10.1038/nature11174 22722859 PMC3738909

[ref-22] Mourier T Carret C Kyes S: Genome-wide discovery and verification of novel structured RNAs in *Plasmodium falciparum*. *Genome Res.* 2008;18(2):281–292. 10.1101/gr.6836108 18096748 PMC2203626

[ref-23] Neafsey DE Galinsky K Jiang RH: The malaria parasite *Plasmodium vivax* exhibits greater genetic diversity than *Plasmodium falciparum*. *Nat Genet.* 2012;44(9):1046–1050. 10.1038/ng.2373 22863733 PMC3432710

[ref-24] Otto TD Böhme U Sanders M: Long read assemblies of geographically dispersed *Plasmodium falciparum* isolates reveal highly structured subtelomeres [version 1; peer review: 3 approved]. *Wellcome Open Res.* 2018;3:52. 10.12688/wellcomeopenres.14571.1 29862326 PMC5964635

[ref-25] Otto TD Rayner JC Böhme U: Genome sequencing of chimpanzee malaria parasites reveals possible pathways of adaptation to human hosts. *Nat Commun.* 2014;5: 4754. 10.1038/ncomms5754 25203297 PMC4166903

[ref-26] Otto TD Sanders M Berriman M: Iterative Correction of Reference Nucleotides (iCORN) using second generation sequencing technology. *Bioinformatics.* 2010a;26(14):1704–1707. 10.1093/bioinformatics/btq269 20562415 PMC2894513

[ref-27] Otto TD Wilinski D Assefa S: New insights into the blood-stage transcriptome of *Plasmodium falciparum* using RNA-Seq. *Mol Microbiol.* 2010b;76(1):12–24. 10.1111/j.1365-2958.2009.07026.x 20141604 PMC2859250

[ref-28] Pain A Böhme U Berry AE: The genome of the simian and human malaria parasite *Plasmodium knowlesi*. *Nature.* 2008;455(7214):799–803. 10.1038/nature07306 18843368 PMC2656934

[ref-29] Painter HJ Chung NC Sebastian A: Genome-wide real-time *in vivo* transcriptional dynamics during *Plasmodium falciparum* blood-stage development. *Nat Commun.* 2018;9(1): 2656. 10.1038/s41467-018-04966-3 29985403 PMC6037754

[ref-30] Raabe CA Sanchez CP Randau G: A global view of the nonprotein-coding transcriptome in *Plasmodium falciparum*. *Nucleic Acids Res.* 2010;38(2):608–617. 10.1093/nar/gkp895 19864253 PMC2811010

[ref-31] Rutledge GG Böhme U Sanders M: *Plasmodium malariae* and *P. ovale* genomes provide insights into malaria parasite evolution. *Nature.* 2017;542(7639):101–104. 10.1038/nature21038 28117441 PMC5326575

[ref-32] Swain MT Tsai IJ Assefa SA: A post-assembly genome-improvement toolkit (PAGIT) to obtain annotated genomes from contigs. *Nat Protoc.* 2012;7(7):1260–1284. 10.1038/nprot.2012.068 22678431 PMC3648784

[ref-33] Tachibana SI Sullivan SA Kawai S: *Plasmodium cynomolgi* genome sequences provide insight into *Plasmodium vivax* and the monkey malaria clade. *Nat Genet.* 2012;44(9):1051–1055. 10.1038/ng.2375 22863735 PMC3759362

[ref-34] Tang Y Meister TR Walczak M: A mutagenesis screen for essential plastid biogenesis genes in human malaria parasites. *PLoS Biol.* 2019;17(2):e3000136. 10.1371/journal.pbio.3000136 30726238 PMC6380595

[ref-35] The Gene Ontology Consortium: Expansion of the Gene Ontology knowledgebase and resources. *Nucleic Acids Res.* 2017;45(D1):D331–D338. 10.1093/nar/gkw1108 27899567 PMC5210579

[ref-36] Vembar SS Seetin M Lambert C: Complete telomere-to-telomere *de novo* assembly of the *Plasmodium falciparum* genome through long-read (>11 kb), single molecule, real-time sequencing. *DNA Res.* 2016;23(4):339–351. 10.1093/dnares/dsw022 27345719 PMC4991835

[ref-40] Walliker D Quakyi IA Wellems TE: Genetic analysis of the human malaria parasite *Plasmodium falciparum*. *Science.* 1987;236(4809):1661–1666. 10.1126/science.3299700 3299700

[ref-37] Wilson RJ Denny PW Preiser PR: Complete gene map of the plastid-like DNA of the malaria parasite *Plasmodium falciparum*. *J Mol Biol.* 1996;261(2):155–172. 10.1006/jmbi.1996.0449 8757284

[ref-38] Zhang M Wang C Otto TD: Uncovering the essential genes of the human malaria parasite *Plasmodium falciparum* by saturation mutagenesis. *Science.* 2018;360(6388): pii: eaap7847. 10.1126/science.aap7847 29724925 PMC6360947

