# Supplementary material for: Progression of the canonical reference malaria parasite genome from 2002–2019
Source: Wellcome Open Res. 2019 May 28;4:58. Originally published 2019 Mar 29. [Version 2] doi: 10.12688/wellcomeopenres.15194.2 (PMC6484455; doi:10.12688/wellcomeopenres.15194.2)
Supplement: Peer review history

# Peer review: Reviewer response for version 2

**DOI:** 10.21956/wellcomeopenres.16707.r35630

**Published:** 2019-05-30

**Authors:** Manuel Llinas

**Affiliations:** 1 Department of Biochemistry and Molecular Biology, Penn State University, University Park, PA, USA; 2 Department of Chemistry, Penn State University, University Park, PA, USA; 3 The Huck Center for Malaria Research, Penn State University, University Park, PA, USA

I have read through the manuscript and comments and am fine with the modifications that have been made. In my opinion, this important manuscript can be approved for indexing.

I have read this submission. I believe that I have an appropriate level of expertise to confirm that it is of an acceptable scientific standard.

# Peer review: Reviewer response for version 1

**DOI:** 10.21956/wellcomeopenres.16577.r35172

**Published:** 2019-04-26

**Authors:** Manuel Llinas

**Affiliations:** 1 Department of Biochemistry and Molecular Biology, Penn State University, University Park, PA, USA; 2 Department of Chemistry, Penn State University, University Park, PA, USA; 3 The Huck Center for Malaria Research, Penn State University, University Park, PA, USA

This work by Böhme *et al*. provides a unique summary of a number of iterative improvements that have been made to the *Plasmodium falciparum *reference genome since 2002. In some regards it is a historical review detailing the evolution of this critical genome, highlighting a significant number of gene modifications, losses as well as additions. As described, the current status of the *P. falciparum *genome should be the envy of any field focused on a single model species. The examples of modifications to the genome are adequately depicted throughout and easy to follow. Overall, the exceptional curation of this genome is largely due to the diligent and consistent attention to the quality and completeness that it has received over the years from this team at the Wellcome Sanger Institute. 

 One new feature for the malaria parasitology community is the creation of a “population” reference genome, Pfref1, that accounts for sequence differences between strains. While I applaud this effort since there is a need to encompass the diversity of parasites strains, it is not clear to me whether 3 isolates is sufficient to capture the known major *P. falciparum *haplotypes, as claimed. Perhaps this is true, but do IT, DD2 and Pf3D7 provide sufficient haplotype diversity? If so, can you demonstrate this? And what type of genes account fort the greatest haplotype diversity?

 Also, with Pfref1, how many genes are now included (and nucleotide increase) to map against?

 I think the manuscript could also be somewhat enhanced by describing which sequencing and/or annotation algorithms/approaches have contributed the most significantly to the changes and improvements to the current *P. falciparum *genome overall.

 Although the manuscript describes a large gain in pseudogenes, given recent reports that some pseudogenes may actually express functional proteins ^1^ this should be mentioned as a possible outcome for genes predicted to be non-functional.

 On page 5, it is stated that the number of verified genes has changed from 597 to 1296 genes. It is unclear what “experimentally verified” implies. What verification was applied to these genes?

**Minor comment** **:**

 Please change reference to “AP2 proteins” to ApiAP2 proteins. AP2 refers solely to the DNA binding domain region of these proteins.

 On page 7, three “types” of genes are described (type-1, type-2, and type-3). For the non-experts, please describe what these types refer to?

I have read this submission. I believe that I have an appropriate level of expertise to confirm that it is of an acceptable scientific standard, however I have significant reservations, as outlined above.

## References

[1] Macedo SilvaTDuque AraujoRWunderlichG: The pseudogene SURFIN 4.1 is vital for merozoite formation in blood stageP. falciparum. *bioRxiv*.2019; 10.1101/562124 10.1101/562124

# Response

**Published:** 2019-05-24

**Authors:** Ulrike Böhme

**Affiliations:** Sanger Institute, UK

Thank you for your constructive feedback.

1. One new feature for the malaria parasitology community is the creation of a “population” reference genome, Pfref1, that accounts for sequence differences between strains. While I applaud this effort since there is a need to encompass the diversity of parasites strains, it is not clear to me whether 3 isolates is sufficient to capture the known major *P. falciparum *haplotypes, as claimed. Perhaps this is true, but do IT, DD2 and Pf3D7 provide sufficient haplotype diversity? If so, can you demonstrate this? And what type of genes account fort the greatest haplotype diversity?

Our intention is to provide a reference that will be sufficient to capture the major haplotypes by sequence-mapping approaches. IT, DD2 and Pf3D7 do not represent the full of universe of haplotype diversity. In the paper we state that this supplemented sequence “better represents the pan genome for this species”. Additional sequences can be easily added as they are identified, using this consistent format.

2. Also, with Pfref1, how many genes are now included (and nucleotide increase) to map against?

We found 4 core genes that are present in other isolates.

3. I think the manuscript could also be somewhat enhanced by describing which sequencing and/or annotation algorithms/approaches have contributed the most significantly to the changes and improvements to the current *P. falciparum *genome overall.

As shown in Figure 3, the biggest number of changes were done between 2007 and 2010. This is due to RNA-Seq data and TBLASTX comparison which is described in the methods section: “RNA-Seq data and TBLASTX comparisons were the primary supporting evidence for manual improvements to gene models.”

4. Although the manuscript describes a large gain in pseudogenes, given recent reports that some pseudogenes may actually express functional proteins this should be mentioned as a possible outcome for genes predicted to be non-functional.

We’ve added a sentence to the Table 1 legend to state that pseudogenes have simply been defined operationally as genes with premature stop codons or at least one frameshift.

5. On page 5, it is stated that the number of verified genes has changed from 597 to 1296 genes. It is unclear what “experimentally verified” implies. What verification was applied to these genes?

These are genes that have been mentioned by others in a peer-reviewed paper containing some level of experimental evidence.

**Minor comment:**

1. Please change reference to “AP2 proteins” to ApiAP2 proteins. AP2 refers solely to the DNA binding domain region of these proteins.

AP2 has now been changed to ApiAP2.

2. On page 7, three “types” of genes are described (type-1, type-2, and type-3). For the non-experts, please describe what these types refer to?

This has now been clarified in the text.

“The differences have been incorporated into the reference as three classes ( Figure 5). The first **(type-1)** are “patches” to correct errors in Pf3D7 (version 3.2), for example a missing centromere on chromosome 10 and a missing gene on chromosome 13 ( Figure 5, Figure 6A), the second **(type-2)** are core genes that are present in other sequenced isolates, i.e. P. falciparum IT or P. falciparum DD2 but are missing in Pf3D7 ( Figure 5, Figure 6B) and the third **(type-3)** are dimorphic genes where alternative alleles cannot be mapped to the one currently present in Pf3D7.”

# Peer review: Reviewer response for version 1

**DOI:** 10.21956/wellcomeopenres.16577.r35170

**Published:** 2019-04-25

**Authors:** Anthony A. Holder

**Affiliations:** 1 Francis Crick Institute, London, UK

In the almost 20 years since the publication of the *Plasmodium falciparum* 3D7 genome sequence there have been tremendous improvements in the completeness and accuracy of the sequence and genome structure, as well as an impressive improvement in the annotation. These advances have been achieved by a combination of further sequence analysis of both genome and transcripts, as well as automatic and literature and community-based manual annotation. This paper spells out the patient and often painstaking strategies that have been used since the first publication to ensure that there is as far as possible a complete and fully annotated genome sequence available to the scientific community. Such information is not only essential for much hypothesis-driven research into parasite biology and parasite-host interactions, or for studies of parasite evolution and epidemiology, but also underpins other global approaches, such as proteomics analysis. The work highlights some of the problems that needed to be addressed and provides some examples of the solutions that were found and applied. The ongoing improvements in annotation will facilitate research into specific areas of cell biology and metabolism, and contribute to efforts to translate this knowledge into products useful for interventions to control malaria.

 In addition to the work on the reference cloned 3D7 parasite line, the authors also describe the establishment of an extended data set comprised of a population reference genome of *P. falciparum,* called Pfref1. This will facilitate mapping and comparative studies of isolates and lines that cannot be achieved using the 3D7 reference alone. Such a reference is likely to be very useful for studies of parasite evolution and spread, for example in response to selective pressures. 

 The data are updated regularly and readily available, for example through GeneDB or PlasmoDB and through the documented web sites for further specific details.  

 Minor textual corrections:

1. The last sentence of the abstract needs improvement.
2. In the Methods section, subsection ‘PfRef1 reference genome’, the second to last sentence requires attention.
3. On page 5, second paragraph, the amino acid names isoleucine and valine are not normally capitalized.
4. In Figure 3, during the period 2007-2010, presumably the blue bar should represent 69 new rather than 69 changed.

I have read this submission. I believe that I have an appropriate level of expertise to confirm that it is of an acceptable scientific standard.

# Response

**Published:** 2019-05-24

**Authors:** Ulrike Böhme

**Affiliations:** Sanger Institute, UK

Thank you for your constructive feedback.

Minor textual corrections:

1. The last sentence of the abstract needs improvement.

This sentence has been corrected to:

*“P. falciparum* *3D7 will continue to be maintained, with ongoing curation ensuring continual improvements in annotation quality.”*

2. In the Methods section, subsection ‘PfRef1 reference genome’, the second to last sentence requires attention.

This sentence has been corrected: “ *Each file contains a sequence variant with a 500 bp anchor sequence on either side to enable unambiguous placement along the 3D7 reference.”*

3. On page 5, second paragraph, the amino acid names isoleucine and valine are not normally capitalized.

This has now been changed.

4. In Figure 3, during the period 2007-2010, presumably the blue bar should represent 69 new rather than 69 changed.

Thank you for noticing this. Figure 3 has now been corrected.

# Peer review: Reviewer response for version 1

**DOI:** 10.21956/wellcomeopenres.16577.r35169

**Published:** 2019-04-17

**Authors:** Thomas E. Wellems

**Affiliations:** 1 Laboratory of Malaria and Vector Research, National Institute of Allergy and Infectious Diseases, National Institutes of Health, Rockville, MD, USA

The availability and accuracy of *Plasmodium falciparum* genome information is crucial to molecular and genetic studies of malaria parasites. This manuscript describes valuable updates and improvements to the sequences and annotations of the 14 nuclear chromosomes, apicoplast and mitochondrial sequences of the *P. falciparum* 3D7 clone, including summaries of the progressive assemblies, gap closures, gene structure refinements, and functional annotations. In addition, a *P. falciparum* population database (Pfref1) of parasite variant types is provided for the first time; Pfref1 and its future expansion will facilitate studies of genome regions that are missing or greatly diverged from those of the 3D7 reference.

 Specific comments:

1. In Figure 1, state whether or not coding directions of the gene sequences (orientations) are indicated by positions of the marks above or below the chromosome lines.
2. The numbers of genes in several versions listed in Table 1 appear to differ from the numbers in the corresponding versions of Figure 4. For example, Table 1 lists 5280 genes in Version 3.2 but Figure 4 notes 5438 genes. Check and correct/explain the differences.
3. Figure 2 can be improved and made easier to read by providing the gene IDs directly on the panels. The red blocks between v1 and v3.2 can be much less tall and the coding regions heightened to draw better attention to the gene information.
4. In Figure 4, the 2007, 2009, 2010, 2011 versions are all labeled the same: 2.1.4. However, the 2010 version in Figure 3 is labeled as 2.1.5. Correct/clarify the labels of the different versions.
5. In Table 2, should the “Number of GO annotations (2018)” be corrected to read “Number of GO annotations (2019)”?
6. For the Pfref1 population reference, major sequence variations are included from the three different lab isolates. Please clarify how copy number variations are treated.   
7. Introduction, page 3. *P. falciparum* 3D7 is from the NF54 isolate (Walliker *et al.* 1987, *Science*, footnote 11 ^1^). NF54 has served along with 3D7 as a major focus of malaria research. Inclusion of this information would be helpful to readers, especially as the close relationship of 3D7 to NF54 is often unrecognized or forgotten.

I have read this submission. I believe that I have an appropriate level of expertise to confirm that it is of an acceptable scientific standard.

## References

[1] WallikerDQuakyiIWellemsTMcCutchanTSzarfmanALondonWCorcoranLBurkotTCarterR: Genetic analysis of the human malaria parasite Plasmodium falciparum. *Science*.1987;236(4809) : 10.1126/science.3299700 1661-1666 10.1126/science.3299700 3299700

# Response

**Published:** 2019-05-24

**Authors:** Ulrike Böhme

**Affiliations:** Sanger Institute, UK

Thank you for your constructive feedback.

Specific comments:

1. In Figure 1, state whether or not coding directions of the gene sequences (orientations) are indicated by positions of the marks above or below the chromosome lines.

Thank you for this suggestion. This information has now been added in the figure legend.

*“Genes above the chromosome lines are located on the forward strand, genes below the chromosome lines are on the reverse strand.”*

2. The numbers of genes in several versions listed in Table 1 appear to differ from the numbers in the corresponding versions of Figure 4. For example, Table 1 lists 5280 genes in Version 3.2 but Figure 4 notes 5438 genes. Check and correct/explain the differences.

As stated in the figure legend, Table 1 lists the gene numbers without pseudogenes. Pseudogenes are separately listed in Table 1. Version 3.2 has 5280 protein-coding genes and 158 pseudogenes. Adding both numbers will give you a total of 5438 genes. We’ve now added the following sentence to the legend of Figure 4 to make this clearer.

*“* *The number of genes between October 2002 and March 2019 are compared. The total number of genes includes pseudogenes.”*

3. Figure 2 can be improved and made easier to read by providing the gene IDs directly on the panels. The red blocks between v1 and v3.2 can be much less tall and the coding regions heightened to draw better attention to the gene information.

Thank you for the suggestion to improve this figure. Gene IDs have been added on the panels. The TBLASTX matches are now shown in grey and the height of the TBLASTX matches has been shortened. All genes are shown in red. This is an ACT screenshot which means that coding regions cannot be heightened. But we hope that the other changes will draw more attention to the gene information.

4. In Figure 4, the 2007, 2009, 2010, 2011 versions are all labeled the same: 2.1.4. However, the 2010 version in Figure 3 is labeled as 2.1.5. Correct/clarify the labels of the different versions.

Thank you for noticing this. The number in the legend of Figure 3 has now been changed from 2.1.5 to 2.1.4.

5. In Table 2, should the “Number of GO annotations (2018)” be corrected to read “Number of GO annotations (2019)”?

This has now been corrected.

6. For the Pfref1 population reference, major sequence variations are included from the three different lab isolates. Please clarify how copy number variations are treated.

Most CNVs can be detected and quantified using the strictly 3D7 sequence as a reference (based on mapped coverage of reads). The Pfref1 simply extends the ref to those areas that are missing or highly diverged from 3D7.

7. Introduction, page 3. *P. falciparum*3D7 is from the NF54 isolate (Walliker *et al.* 1987, *Science*). NF54 has served along with 3D7 as a major focus of malaria research. Inclusion of this information would be helpful to readers, especially as the close relationship of 3D7 to NF54 is often unrecognized or forgotten.

We have addressed this with the following text “the genome of *Plasmodium falciparum *3D7 (a clone from the NF54 isolate) …”. We’ve also added the reference.
